# Escenario de riesgo de introducción de la influenza tipo A en México estimado mediante geointeligencia

**DOI:** 10.26633/RPSP.2019.32

**Published:** 2019-03-27

**Authors:** Enrique Ibarra-Zapata, Darío Gaytán-Hernández, Gustavo Mora Aguilera, Miguel Ernesto González Castañeda

**Affiliations:** 1 Universidad Autónoma de San Luis Potosí Universidad Autónoma de San Luis Potosí Facultad de Enfermería y Nutrición México Facultad de Enfermería y Nutrición, Universidad Autónoma de San Luis Potosí, México.; 2 Campus Montecillos Campus Montecillos Colegio de Posgraduados Texcoco México Colegio de Posgraduados, Campus Montecillos, Texcoco, México.; 3 Universidad de Guadalajara Universidad de Guadalajara Departamento de Geografía y Ordenación Territorial México Departamento de Geografía y Ordenación Territorial, Universidad de Guadalajara, México.

**Keywords:** Medición de riesgo, agente patógeno biológico, virus de la influenza A, análisis espacial, México, Risk assessment, noxae, influenza A virus, spatial analysis, Mexico, Medição de risco, agente patogênico biológico, virus da influenza A, análise espacial, México

## Abstract

**Objetivo.:**

Estimar el escenario potencial probabilístico de introducción del agente causal de la influenza tipo A en México mediante geointeligencia sanitaria.

**Métodos.:**

Estudio ecológico en el que consideran 1 973 brotes de influenza con alto grado de patogenicidad en el mundo durante el período 2014-2016. Se desarrolló un modelado geoespacial con herramientas de la geointeligencia, como la representación espacial, modelo de conexidad, caracterización espacial de la fuente de inoculo con el modelo de máxima entropía y la curva característica de operación receptora (COR) mediante la evaluación espacial multicriterio y se validó con el índice de Moran y la regresión geográficamente ponderada.

**Resultados.:**

Se estimaron las isocronas de riesgo sanitario con una distancia de 548 km y su crecimiento exponencial; hasta la cuarta isócrona se identificaron las costas este y oeste de Estados Unidos de América (EEUU) y una porción de América Central como posible superficie que favorece la introducción del patógeno. Se obtuvo, también, una curva COR = 0,923, se identificaron dos períodos de riesgo de introducción (setiembre-marzo) y (abril-agosto) con trayectorias de norte-sur y sur-norte respectivamente, con alta autocorrelación positiva para el modelado geoespacial, y se estimó un escenario donde más de la mitad de México se encuentra en un riesgo alto de introducción, con 78 millones de personas expuestas. Se identificó una asociación positiva entre las áreas de riesgo significativo (*P* < 0,001).

**Conclusión.:**

Se evidencia que más de 50% del territorio mexicano se encuentra en riesgo de introducción del agente causal de la influenza tipo A, con aproximadamente 70% de la población expuesta.

## Abstract (en)

**Objective.:**

Estimate the probabilistic potential of introduction of the causative agent of influenza type A in Mexico, using geo-intelligence applied to health.

**Methods.:**

Ecological study of 1,973 influenza outbreaks with a high degree of pathogenicity, worldwide during the period 2014-2016. Geospatial modeling was developed with geo-intelligence tools such as spatial representation, a relational model, spatial characterization of the inoculum source with the maximum entropy model and the receiver operating characteristic (ROC) curve, using multicriteria spatial analysis. This was validated with the Moran index and geographically weighted regression.

**Results.:**

Isochrones (at an initial distance of 548 km) were estimated for health risks and their exponential growth; at the fourth isochrone, the east and west coasts of the United States of America and a part of Central America were identified as possible areas that favor the introduction of the pathogen. Also, a COR curve = 0.923 was obtained; two risk periods for introduction were identified (September-March and April-August, with north-south and south-north trajectories, respectively) with high positive autocorrelation for geospatial modeling; and in one scenario, more than half of Mexico was found to be at high risk of introduction, with an estimated 78 million people exposed. A positive association was identified between significant risk areas (*p* < 0.001).

**Conclusion.:**

More than 50% of Mexican territory was found to be at risk of introduction of the causative agent of influenza type A, with approximately 70% of the population exposed.

## RESUMO

**Objetivo.:**

Estimar o cenário probabilístico em potencial de introdução do vírus da influenza A no México com o uso de inteligência geográfica em saúde.

**Métodos.:**

Estudo ecológico de 1.973 surtos mundiais de influenza de alta patogenicidade ocorridos no período 2014–2016. Foi desenvolvido um modelo geoespacial com ferramentas de inteligência geográfica, como representação espacial, modelo de conexidade, caracterização espacial da fonte de inóculo com o modelo de máxima entropia e curva ROC (receiver operating characteristic) com avaliação espacial por múltiplos critérios e validação com o índice de Moran e regressão geograficamente ponderada.

**Resultados.:**

Foram estimadas isócronas do risco de saúde com uma distância de 548 km e o crescimento exponencial destes linhas; até a quarta isócrona, foram identificadas as costas leste e oeste dos Estados Unidos (EUA) e parte da América Central como possível superfície que favorece a introdução do vírus. Foi também estimada uma curva ROC de 0,923, sendo identificados dois períodos de risco de introdução do vírus (setembro–março e abril–agosto) com trajetórias de norte-sul e sul-norte, respectivamente, com elevada autocorrelação positiva para o modelo geoespacial. Foi estimado um cenário em que mais da metade do México apresenta alto risco de introdução do vírus da influenza, com 78 milhões de pessoas expostas. E foi observada uma associação positiva entre as áreas de risco significativo (*P* < 0,001).

**Conclusão.:**

Observa-se que mais de 50% do território mexicano está sob risco de introdução do vírus da influenza A, com cerca de 70% da população exposta.

La influenza tipo A es una enfermedad infecciosa causada por un virus de la familia *Orthomyxoviridae* y se clasifica en de baja patogenicidad y de alta patogenicidad (1). En todo el mundo, algunas cepas del virus de la influenza tipo A pueden causar altas tasas de mortalidad. En 2017, la Organización Mundial de la Salud (OMS) publicó los primeros reportes de serotipos con alto grado de patogenicidad (H5N1 y H7N9), capaces de afectar a los seres humanos y con tasas de mortalidad de 27% y 60%, respectivamente (2). Lo alarmante desde una perspectiva epidemiológica es que, en ambos casos, existe gran capacidad de mutación y con ello la posibilidad de generar nuevas recombinaciones de las que no se encontró evidencia.

En este sentido, el agente causal de la influenza tipo A se considera un grave problema para la salud mundial, ya que pueden existir hasta 144 recombinaciones posibles según las hemaglutininas y neuraminidasas de superficie que las componen (3). Por otra parte, el riesgo se potencializa por la existencia de múltiples hospederos y los mecanismos de dispersión de este tipo de virus que, en forma implícita, otorga un gran potencial pandémico.

Se la ha catalogado también como una enfermedad trasfronteriza, ya que ha cruzado la barrera entre las especies animal y humana (4). Según la OMS, en años recientes han existido brotes de alta patogenicidad y de baja patogenicidad en varias regiones del mundo, donde se considera una enfermedad compleja con graves repercusiones en la salud de la población en Europa, América, Asia y Oriente Medio (5-7). Por otra parte, ha tenido un impacto grave en la economía y la salud pública en regiones donde se consideran endémicos virus con alto grado de patogenicidad (8).

Se dice que de cinco nuevas enfermedades humanas que aparecen cada año en el mundo, tres son de origen animal y que al menos 75% de los agentes patógenos de enfermedades infecciosas reemergentes del ser humano son de origen zoonótico, como las variantes del agente causal de la influenza tipo A (9).

Por lo anterior, las enfermedades reemergentes representan un riesgo global para la salud pública, puesto que existen reservorios y mecanismos de dispersión que favorecen su propagación. El dinamismo del mundo actual con la globalización, el cambio climático y el comportamiento humano multiplica las oportunidades para que los patógenos colonicen nuevos territorios y evolucionen bajo nuevas formas o serotipos (9, 10), los cuales se pueden convertir en riesgos potenciales para la salud humana.

Entre octubre de 2014 y diciembre de 2016 se reportó la existencia de 1 973 brotes de influenza con alto grado de patogenicidad en el mundo, con dictámenes positivos avalados por laboratorios de la OMS (1) y la Organización Mundial de Sanidad Animal (OIE, por sus siglas en francés), (9) cuya distribución geográfica abarcaba el sudeste asiático, Oriente Medio, África central y brotes dispersos en Asia, Europa, norte de África y América del Norte.

La complejidad de los problemas en salud pública en el mundo actual hace que se perciban como un poliedro conformado por múltiples y cambiantes perspectivas, lo que justifica la variedad de enfoques con las que se deben abordar dichas problemáticas (11). Este es el caso del análisis y modelado geoespacial, donde se aborda el riesgo de introducción que representa el agente causal de la influenza con alto grado de patogenicidad ya que, ante un brote, la situación en salud puede modificarse con rapidez y traer consecuencias negativas, lo que implica una evaluación y acciones inmediatas (12).

Por ello, incorporar enfoques alternativos podría coadyuvar en la forma de abordar, comprender y atender este tipo de enfermedades (13), a través de la modelación de las posibles áreas de riesgo de introducción de un patógeno hacia un territorio determinado y evidenciar el riesgo sanitario con base en el contexto espacio-temporal. Por otra parte, no se encontraron estudios que hayan considerado las distancias euclidianas en la estimación del escenario de potencial probabilístico de introducción (Ppi), que se incorporan en el presente estudio para robustecer la metodología.

Las aplicaciones de estas metodologías pueden ayudar en el diseño de estrategias hacia áreas específicas y políticas direccionadas; con ello se lograrían mejores resultados y se optimizarían los recursos.

Derivado de lo anterior, el objetivo de la presente investigación fue estimar el escenario de potencial probabilístico de introducción (Ppi) del agente causal de la influenza tipo A en México mediante métodos y técnicas de carácter geoespacial.

## MATERIALES Y MÉTODOS

Para el desarrollo del modelo se utilizó el software QGIS Versión 2.18 Las Palmas de Gran Canarias® y ArcGis-ArcMap versión 10.1^®^.

El modelado geoespacial se sustenta en el enfoque de la geointeligencia, que se refiere a la explotación y análisis avanzado de datos obtenidos por sistemas de colección de información geoespacial bajo un enfoque transdisciplinario dirigido a los tomadores de decisiones (14). Desde el punto de vista epidemiológico, se apoya en el concepto de “una sola salud” que señala que la salud humana y la sanidad animal son interdependientes y están vinculadas a los ecosistemas en los cuales coexisten (9).

Es fundamental incorporar una visión integradora mediante el análisis exploratorio de datos espaciales (AEDE), ya que los hallazgos proporcionan información al tomador de decisiones en salud pública, para prestar atención adicional a la planificación espacial de medidas de control ante enfermedades reemergentes (15, 16).

El presente estudio es ecológico cuantitativo con información geográficamente referida, la cual se considera el insumo principal en la estimación del escenario de Ppi. Como universo se consideraron 1 973 brotes distribuidos en 45 países, entre ellos Canadá, EE.UU. y México, ocurridos entre 2014-2016, incluidos los ocho serotipos de influenza con alto grado de patogenicidad (H5, H5N1, H5N2, H5N5, H5N6, H5N8, H5N9 y H7N9) (5), así como los factores relacionados (7).

El modelado geoespacial considera como eje rector el concepto de contagio que involucra el número de eventos espaciales, la intensidad de la cepa, el análisis epidemiológico y el análisis espacial, que se integran y desarrollan en cinco etapas.

### Primera etapa

**FIGURA 1:**
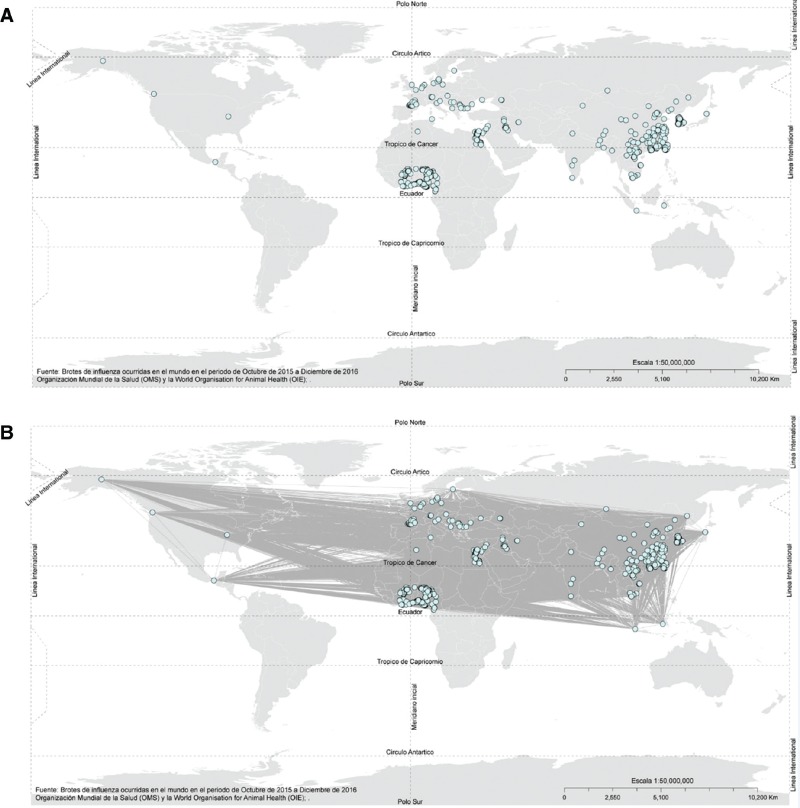
Representación espacial y modelo de conexidad de las detecciones de influenza con alto grado de patogenicidad en el mundo en el período 2014-2016

Se realizó la representación espacial de la ubicación de los brotes en un sistema de coordenadas, ya que la agrupación de brotes puede representar poblaciones en riesgo (10). Por ello, se lo definió como el insumo detonante para estimar el escenario de Ppi (figura 1a).

### Segunda etapa

Se desarrolló un modelo de conexidad de los brotes (figura 1b), que estima las medidas de tendencia central y de dispersión** para la delimitación territorial de superficies a través de la denomina distancia general (Dg). Se considera el territorio de México como nodo de interés y la asociación espacial con las múltiples detecciones en el período de estudio integradas con la siguiente ecuación:

**Dg** = *(((∑((dni/nO1)+(dni/nO2)+(dni/nO3)+...+…+(dni/nO1972)+(dni/nO1973))+(dnO1/nO1)+(dnO1/nO2)+(dnO1/nO3)+...+…+(dnO1/nO1972)+(dnO1/nO1973))+((dnO1973/nO1)+(dnO1973/nO2)+(dnO1973/nO3)+...+…+(dnO1973/nO1972)+ (dnO1973/nO1973)/N))/N))).*

Donde:

Dg: distancia general.

d: distancia.

ni: nodo de interés.

nO: nodo de origen.

N: total de detecciones.

***Fuente:*** elaboración propia con información de la Organización Mundial de la Salud y la Organización Mundial de Sanidad Animal (2, 9).

De esta forma, se estimaron los insumos para el cálculo de las isocronas de riesgo sanitario (Is-RS) (11), con base en la técnica de estadística clásica de análisis de la relación *(σ′*^2^*/µ)* (varianza/media) (17) con crecimiento exponencial. Mediante la denominada área de influencia (5), se consideró una media de 6 825 km y desviación estándar de 6 277 km obtenidos del modelo de conexidad como insumos para el cálculo con la siguiente ecuación:

$${\text{Is-RS = ((d}}^{\text{med}}{\text{-desvest)}}^{\text{x}}\text{)}$$

Donde:

Is-RS: isocronas de riesgo sanitario.

d^med^: distancia media.

desvest: desviación estándar.

x: coeficiente exponencial.

**FIGURA 2:**
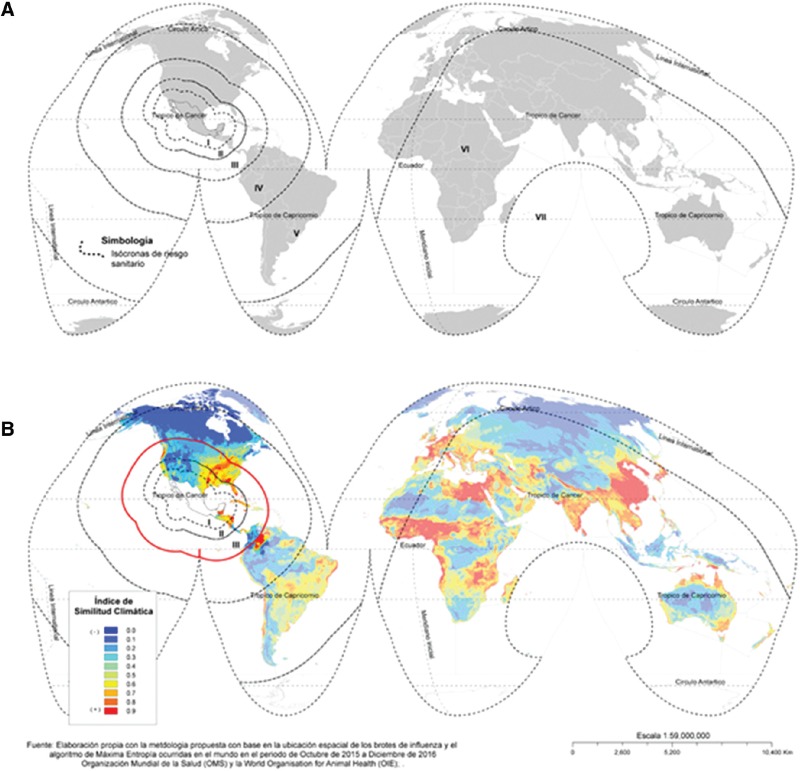
Isocronas de riesgo sanitario y modelo de similitud climática del agente causal de la influenza con alto grado de patogenicidad en el período 2014-2016

Desde el punto de geoestadístico, el modelo crea una red de conectividad entre los nodos; es decir, establece una distancia media ponderada como una imaginaria referencial, de la cual se obtiene una Is-RS inicial de 548 Km y su crecimiento a seis Is-RS. Estas últimas describen la continuidad espacial vista como un fenómeno territorial e involucra funciones para modelar la variación territorial y, con ello, interpolar en el espacio en el valor del área de influencia (18, 19) en el denominado espacio euclidiano (figura 2a).

### Tercera etapa

Involucró la caracterización del área de fuente de inoculo para entender la ecología viral del agente causal de la influenza tipo A (5) mediante el modelo de máxima entropía (Maxent) que, según diferentes autores (20, 21), es robusto para estimar el nicho ecológico (áreas de confort) de distintas especies. Además, estima y utiliza la curva característica de operación receptora (COR) para evaluar la estimación el diagnóstico que este causa, así como el peso que cada variable representa.

***Fuente:*** elaboración propia con información de la Organización Mundial de la Salud y la Organización Mundial de Sanidad Animal (2, 9).

El Maxent^®^ ha sido de gran utilidad en la estimación de escenarios de la influenza de alta patogenicidad en el sudeste asiático y otras regiones del planeta (22, 23). Posee gran efectividad en estimar áreas semejantes desde el punto de vista ambiental (22, 24), mediante la asociación espacial de 19 variables ambientales con criterios biológicamente significativos de la base mundial (BIOCLIM) con una resolución espacial de 0,5 grados (25), lo que permite definir las áreas de confort para la especie y se describen de la siguiente forma:

BIO1 = temperatura media anual

BIO2 = intervalo diurno medio

BIO3 = isotermalidad (BIO2 / BIO7) (*100)

BIO4 = estacionalidad de la temperatura (desviación estándar * 100)

BIO5 = temperatura máxima del mes más cálido

BIO6 = temperatura mínima del mes más frío

BIO7 = rango anual de temperatura

BIO8 = temperatura media del trimestre más húmedo

BIO9 = temperatura media del trimestre más seco

BIO10 = temperatura media del trimestre más cálido

BIO11 = temperatura media del trimestre más frío

BIO12 = precipitación anual

BIO13 = precipitación del mes más húmedo

BIO14 = precipitación del mes más seco

BIO15 = estacionalidad de la precipitación

BIO16 = precipitación del trimestre más húmedo

BIO17 = precipitación del trimestre más seco

BIO18 = precipitación del trimestre más cálido

BIO19 = precipitación del trimestre más frío

Además, el modelo involucró criterios de exclusión espacial como la duplicidad en la ubicación y la falta de exactitud de los brotes o detecciones (se excluyeron los datos con menos de cuatro dígitos después del punto).

**CUADRO 1:** Variables y pesos asignados para estimar el potencial probabilístico de introducción del agente causal de la influenza tipo A mediante la evaluación espacial multicriterio

Variable y relación	Definición operacional	Técnica de asignación de pesos	Pesos para la EEMC	Rangos en la representación de Ppi
Área de influencia (topología de polígono)	Área de riesgo en la introducción del agente causal de la influenza	$S=\sum_{i=1}^{n}w_{i}x_{i}$	0,3	
Fuente de inoculo (topología de polígono)	Superficies caracterizadas según la aptitud ambiental para el agente causal de los virus de la influenza	Donde: S = potencial probalístico en una escala de 0,0 a 0,9	0,3	Muy alto (0,8-0,9) Alto (0,6-0,7) Medio (0,4-0,5)
Rutas de aves migratorias (nodo o punto y línea o red)	Mecanismo de dispersión definidos en dos periodos espacio temporalmente caracterizados.	w_i_ = valor de importancia del factor//subfactores i x_i_ = factor i estandarizado con las funciones de membresía	0,4	Bajo (0,2-0,3) Muy bajo (0,0-0,1)

EEMC; evaluación espacial multicriterio; Ppi, potencial probablístico de introducción.

***Fuente:*** elaboración propia en base a la aptitud de las variables.

### Cuarta etapa

Involucró la caracterización de los mecanismos de dispersión en el área fuente de inóculo apoyado en la hipótesis de que uno de los principales componentes que favorecen la dispersión. Se asocia a las rutas de aves migratorias (6, 8, 26), pues estas son transmisoras secundarias de virus con alto grado de patogenicidad y reservorios primarios de virus de influenza aviar de baja patogenicidad (27).

Por lo anterior, se incorporaron como una variable espacio-temporal y se tomó como referencia los dos períodos de setiembre a marzo con trayectorias de norte a sur y de abril a agosto con trayectorias de sur a norte (28), a partir de los cuales se realizó un proceso de sobreposición geométrico cartográfico. Además, desde el punto de vista de la altitud, se identifican distancias de 10 a 700 metros sobre el nivel de suelo, que representan el rango de altitudes donde se observa la mayor parte de la migración de aves en estudios con radar (29).

### Quinta etapa

Se realizó la integración de las variables en la estimación del Ppi mediante la evaluación espacial multicriterio (EEMC) que involucra la asignación de pesos a través de una matriz de valoración con base en una escala de valores de 0,0 a 0,9. El análisis de diferencias espaciales se realiza mediante la relación nodo/punto, línea/red y topología de polígono, según los criterios epidemiológicos del agente causal de la influenza bajo la siguiente ecuación:

Ppi = (interacción espacial [área de influencia y fuente de inóculo y rutas de aves migratorias])

Luego, mediante el uso del operador boleano espacial AND (para cumplir dos o más condiciones en forma simultánea), se analizó la geometría lógica del riesgo de introducción y se caracterizó el territorio nacional en los rangos Muy alto, Alto, Medio, Bajo y Muy bajo (cuadro 1).

## RESULTADOS

En la (figura 1a) se muestra la representación espacial de los brotes obtenidos en la primera etapa, donde se evidencia la agrupación o los *clusters* en el sudeste asiático, Oriente Medio, Europa y África Central; así como brotes aislados en el continente americano.

En la segunda etapa, se evidencian las áreas de fuente de inóculo mediante las cuatro primeras Is-RS, que aluden al principio geográfico de proximidad bajo la primera ley de Tobler que señala que “todas las cosas están relacionadas entre sí, pero las cosas más próximas en el espacio tienen una relación mayor que las distantes”; así, se caracteriza un área de influencia de 2 192 km denominada área de fuente de inóculo (figura 2a).

En la tercera etapa, se obtuvo el modelo de similitud climática para caracterizar el área de fuente de inoculo en la estimación del Ppi. Este permitió definir las superficies con mayor aptitud ambiental para virus con alta patogenicidad (tonalidades rojas) que, desde el punto de vista territorial, pueden influir en el riesgo de introducción a México. Además, es considerado un modelo robusto, puesto que cuenta con análisis de COR con un valor de 0,923 y supera el valor crítico de 0,700 en este tipo de modelos.

Por otra parte, se identifican las variables principales para la estimación del escenario: la precipitación del trimestre más húmedo contribuye con 22,5%, la temperatura media anual con 21,3%, la estacionalidad de temperatura con 11,6%, la precipitación del trimestre más cálido con 7,8%; y u36,8% se distribuye en las 15 variables ambientales restantes (figura 2b).

Por último, en la cuarta etapa el riesgo de introducción se identificó como área de fuente de inóculo la costa oeste de los EEUU y de mayor extensión la costa este hacia la mitad de ese país, donde se identifica un alto índice de similitud climática con valores entre 0,6 y 0,9 en tonalidades de amarillo a rojo.

**FIGURA 3:**
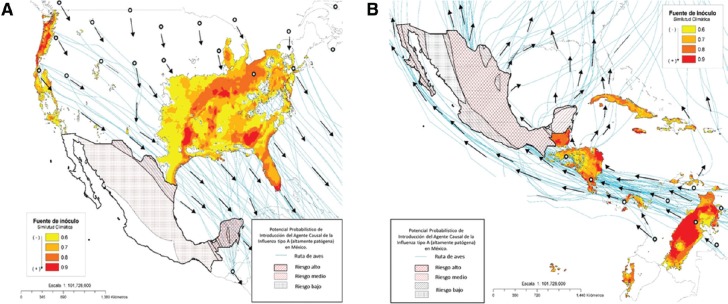
Riesgo de introducción del agente causal de la influenza tipo A con alto grado de patogenicidad en México

Para México se evidencian cuatro porciones en riesgo sanitario, con muy alto riesgo se encuentra la mayor parte de la península de Yucatán y una pequeña porción al norte de Coahuila; con menor superficie, pero alto riesgo se encuentra una pequeña franja que comprende desde la porción central de Chiapas hasta Tabasco, así como la porción norte de los estados de Tamaulipas, Nuevo León, Coahuila y Chihuahua. Sin embargo, casi la mitad del territorio nacional desde la porción más estrecha de Sonora hacia el centro del país se encuentra con un riesgo de introducción alto se encuentra, siguiendo la planicie costera del Golfo, pasando por el istmo de Tehuantepec hasta la frontera sur en Chiapas. En riesgo bajo se encuentra la península de Baja California y parte de la costa del Pacífico, desde Guerrero hasta el norte de Nayarit (figura 3a).

De igual forma, se identificó la región de América Central y El Caribe como un área fuente de inóculo, donde el modelo de similitud climática cuenta con valores que oscilan entre 0,6 y 0,9 con aptitud ambiental que favorece a este tipo de virus.

***Fuente:*** elaboración propia con base en el modelo geoespacial.

Se evidencia con muy alto riesgo la región que abarca desde Chiapas siguiendo el istmo de Tehuantepec por la planicie costera del Golfo hasta el límite norte del país desde Tamaulipas hasta Chihuahua, parte de la península de Baja California y el norte de la península de Yucatán.

Con riesgo medio se encuentra una sección de la península de Baja California, así como una franja de norte a sur de Chihuahua a Durango. Por último, con riesgo bajo se halla la mayor parte de la península de Yucatán y una franja de Sonora a Nayarit (figura 3b).

La estimación del escenario de Ppi del agente causal de la influenza tipo A evidencia que la mayoría de la población 78 millones de habitantes se encuentra con un índice de introducción alto, que se distribuye en más de la mitad del territorio nacional (50,59%). Desde el punto de vista estacional, el mayor riesgo se identifica en el período de setiembre a marzo.

Aproximadamente 24 millones de habitantes tienen un índice de Ppi medio, y se distribuyen en 26,14% de la superficie de México. Sin embargo, con Ppi muy alto se encuentran 5,2 millones de habitantes distribuidos en 8,66 % del territorio nacional. Esto muestra que 94% de la población se encuentra en riesgo de medio a muy alto, lo que implica que 85% de la superficie del país está expuesto a la introducción del agente causal de la influenza tipo A.

**FIGURA 4:**
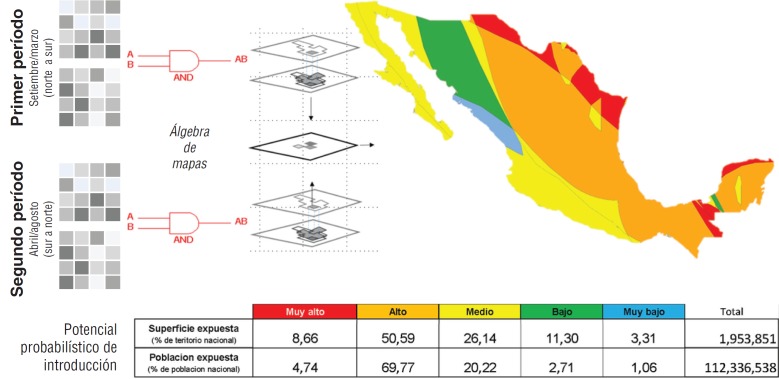
Potencial probabilístico de Introducción del agente causal de la influenza altamente patógena en México

Por último, con un índice de Ppi bajo se encuentran tres millones de habitantes en 11,30 % de la superficie nacional, y con un índice de Ppi muy bajo hay 1,2 millones habitantes (figura 4).

***Fuente:*** Elaboración propia con base en el modelo geoespacial

## DISCUSIÓN

Se identificó que 60% del territorio mexicano se encontraría en riesgo alto y muy alto, lo que refleja que 74,5% de la población se encontraría expuesta a la introducción de virus muy patógeno, con mayor riesgo de setiembre a marzo con una trayectoria sur-norte. Se observa también un área de influencia de 2 192 km, considerada el área fuente de inóculo. En ella se jerarquizaron las subáreas de acuerdo al Ppi. Esta estimación del modelo se validó con el análisis COR del Maxent; el resultado fue de 0,923 superior al valor crítico 0,700 (5), por lo cual se considera un modelo óptimo que indica que el escenario estimado identificó 92,3% de las áreas que cuentan con mayor probabilidad de introducción de virus con alta patogenicidad.

Además, el modelo identificó que las variables temperatura y precipitación fueron las que más influyeron en la estimación del nicho ecológico, lo que permitió la ubicación espacial de las superficies con las condiciones ambientales propicias para la sobrevivencia y desarrollo de este tipo de virus, con alta autocorrelación espacial (30).

Las técnicas y métodos empleados en esta investigación son recomendables para analizar problemas epidemiológicos y sanitarios, ya que pueden estimar una posibilidad de ocurrencia con base en la interacción de variables geofísicas, antrópicas y epidemiológicas y reflejar los resultados en tiempo y espacio. Así, permiten robustecer la toma de decisiones ante problemas remergentes de salud, pues se integran varios factores que potencializan la introducción y movilidad de patógenos (clima, mecanismo de dispersión y hospedantes) de características específicas para subsistir y desarrollarse.

La elaboración de las Is-RS como insumo en la estimación del Ppi permite tener un referente espacial de la distancia representativa entre los brotes de influenza muy patógena; es decir, se incorpora el espacio euclidiano en la caracterización del riesgo sanitario, ya que todo problema en salud es ubicable y diferenciado desde el punto de vista territorial. Esto permite poner límites o áreas de posible alcance del elemento de riesgo, los cuales se potencializan asociados a los procesos de globalización (movimientos trasnacionales) y fenómenos macroambientales como el calentamiento global, que inciden en la aparición de enfermedades remergentes como la influenza con alto grado de patogenicidad. En este sentido, es pertinente señalar que no se encontraron estudios anteriores donde se hayan considerado las distancias euclidianas en la metodología para estimar escenarios de Ppi, lo que puede suponer que la estimación en este estudio sea más robusta, ya que estas fueron incluidas.

Las epidemias de influenza no están determinadas solo por elementos biológicos (16). Por ello, en el presente estudio, la caracterización del Ppi involucró un componente temporal con mayor definición, a diferencia de otras investigaciones sobre riesgo de influenza en los que solo se han utilizado variables bioclimáticas, geográficas, antropógenas o el Maxent^®^ como modelo de distribución potencial (4-6). Nuestros resultados evidencian dos períodos de riesgo de introducción (28), lo que permitió estimar un escenario de introducción con mayor resolución espacial y temporal. Así, se obtuvo un modelo más robusto que muestra el tiempo de mayor riesgo (de setiembre a marzo), con una posible movilización del agente causal de la influenza tipo A de sur a norte. A su vez, esto permitió fortalecer la estimación del riesgo de introducción en el escenario de Ppi a México.

Este tipo de modelos puede fortalecer la toma de decisiones (17) en materia de prevención y actuación ante problemas remergentes de salud como lo es el agente causal de la influenza tipo A. Esto es gracias a que se concibió el Ppi como una herramienta cartográfica que puede coadyuvar a direccionar las acciones y control ante la aparición de brotes o detecciones de este tipo de virus en cualquier lugar del territorio mexicano. Así, es posible fortalecer la respuesta inmediata de los servicios sanitarios y direccionar estrategias que impacten en la vigilancia, monitorización o control de la influenza con alto grado de patogenicidad y coadyuvar en la planificación de estrategias de prevención y actuación ante un brote o detección.

## CONCLUSIONES

El modelado geoespacial constituye una herramienta para la priorización y planificación de acciones y para dar soporte en la toma de decisiones en la salud pública, debido a que representan el riesgo sanitario desde los puntos de vista epidemiológico, espacial y temporal ante enfermedades reemergentes como el agente causal de los virus de influenza tipo A. Se vislumbra un escenario probabilístico de alarma, ya que más de la mitad del territorio mexicano se encontraría en algún nivel de riesgo de tener contacto con virus con alto grado de patogenicidad y una posible combinación con los de baja patogenicidad.

## Contribución de los autores.

EIZ, DGH, GMA y MEGC participaron en la conceptualización, planteo, análisis e interpretación. Todos los autores aprueban la versión final del escrito y asumen la responsabilidad pública por su contenido.

## Financiamiento.

La presente investigación se llevó a cabo con el subsidio otorgado por una beca del Consejo Nacional de Ciencia y Tecnología (CONACYT) en el período 2016-2018.

## Declaración.

Las opiniones expresadas en este manuscrito son responsabilidad del autor y no reflejan necesariamente los criterios ni la política de la *RPSP/PAJPH* y/o de la OPS.

## References

[1] 1. Organización Panamericana de la Salud (OPS). Influenza aviar. Disponible en: https://www.paho.org/hq/index.php?option=com_content&view=article&id=7030%3A2012-avian-influenza&catid=4553%3Aavian-influenza&Itemid=39532&lang=es Acceso el 15 de noviembre de 2017.

[2] 2. Organización Mundial de la Salud. Virus de la gripe aviar y otros virus de la gripe de origen zoonótico. Nota descriptiva, 2016. Disponible en: http://www.who.int/mediacentre/factsheets/avian_influenza/es/ Acceso el 15 de agosto de 2017.

[3] 3. Jiménez Clavero MA. La gripe y sus virus (y II). Virus emergentes y cambio global. Blog de divulgación sobre virus emergentes y las enfermedades que producen, en un contexto de cambio global. 2013. Disponible en: http://www.madrimasd.org/blogs/virusemergentes/2013/05/la-gripe-y-sus-virus-y-ii/ Acceso el 3 de setiembre de 2017.

[4] 4. Zhang Z, Dongmei CH, Yue CH, Tilman MD, Vaillancourte JP, Wenbao L. Risk signals of an influenza pandemic caused by highly pathogenic avian influenza subtype H5N1: spatio-temporal perspectives. Vet J. Disponible en: 10.1016/j.tvjl.2011.08.012 21944318

[5] 5. Keiko AH, Falk H, Lindgren MA. A global model of avian influenza prediction in wild birds: the importance of northern regions. Vet Res. 2013;44:42. Doi.org/10.1186/1297-9716-44-42 Acceso el 12 de diciembre de 2017. 10.1186/1297-9716-44-42 PMC3687566 23763792

[6] 6. Kim B, Marius G, Pfeiffer DU. Modeling habitat suitability for occurrence of highly pathogenic avian influenza virus H5N1 in domestic’s poultry in Asia: a spatial multicriteria decision analysis approach. Spat Spatiotemporal Epidemiol. 2013(4):1-14. 10.1016/j.sste.2012.11.002 23481249

[7] 7. Smallman-Raynor M, Cliff AD. The geographical spread of avian influenza A (H5N1): panzootic transmission (December 2003–May 2006), pandemic potential, and implications. Ann Am Assoc Geogr. 2008;98(3):553-82.

[8] 8. Mohammad A, Hijimans RJ, Abdullah AE, Martinez Lopez B, Perea AM. The use of spatial an spatiotemporal modelling for surveillance of H5N1 highly pathogenic avian influenza in poultry in the Middle East. BioOne Research Evolved. Avian Dis. 2016;60(Ss):146-55. DOI:10.1637/11106-04115-Reg 10.1637/11106-042115-Reg 27309050

[9] 9. Organización de las Naciones Unidas para la Alimentación y la Agricultura/Organización Mundial de la Sanidad Animal (FAO-OIE), 2016. Una sola salud. http://www.oie.int/es/para-los-periodistas/onehealth-es/ Acceso el 8 de julio de 2016.

[10] 10. Smietanka K, Bocian L, Meissner W, Zietek-Barszcz A, Zolkos K. Assessment of the potential distance of dispersal of high pathogenicity avian influenza virus by wild mallards. Avian Dis. 2016;60(S1):316-21. DOI: 10.1637/11080-040715-RegR 10.1637/11080-040715-RegR 27309073

[11] 11. Figueroa de López S. Introducción a la salud pública. Fase 1 Área de Salud Pública, Facultad de Ciencias Médicas, Universidad de San Carlos. Disponible en: https://saludpublicayepi.files.wordpress.com/2012/06/documento-3er-parcial-compilacion-4-documentos.pdf Acceso el 10 de setiembre de 2016.

[12] 12. Organización Panamericana de la Salud (OPS). Evaluación rápida de riesgos de eventos agudos de salud pública, 2015. Disponible en: https://www.paho.org/hq/dmdocuments/2015/2015-cha-evaluacion-rapida-riesgos-eventos.pdf Acceso el 15 de setiembre de 2016.

[13] 13. Tirado F, Gomez A, Rocamora V. The global condition of epidemics: panoramas in A (H1N1) influenza and their consequences for One World One Health Programme. Soc Sci Med. 2015;129:113-22. DOI: 10.1016/j.socscimed.2014.09.003 10.1016/j.socscimed.2014.09.003 PMC7125774 25218795

[14] 14. Consejo Nacional de Ciencia y Tecnología (CONACYT)-CentroGEO. Raising geospatial analysis to geoIntelligence. Latin America Geoespatial Forum, 2015. Disponible en: http://lagf.org/2014/ppt/Rep%20from%20CenterGeo.pdf Acceso el 8 de julio de 2016.

[15] 15. Alimi TO, Fuller DO, Herrera SV, Arevalo-Herrera M, Quinones ML, Stoler JB. A multi-criteria decision analysis approach to assessing malaria risk in northern South America. BMC Public Health. 2016;16:221. 10.1186/s12889-016-2902-7 PMC4778356 26940004

[16] 16. Nallar R, Papp Z, Leighton FA, Epp T, Pasick J, Soos C. Ecological determinants of avian influenza virus, West Nile virus, and avian paramyxovirus infection and antibody status in blue-winged teal (Anas Discors) in the Canadian prairies. J Wild Dis. 2016;52(1):33-46. DOI: 10.7589/2013-07-191 10.7589/2013-07-191 26540179

[17] 17. Díaz Viera MA, Casar González R. Geoestadística aplicada. Universidad Nacional Autónoma de México. Disponible en: http://mmc2.geofisica.unam.mx/cursos/gest/Presentaciones/CG1_2009.pdf Acceso el 16 de diciembre de 2016.

[18] 18. Castillo Ramiro JJ, Gamma LL, Zequeira Larios C. Análisis de regresión lineal en un sistema de información geográfico para determinar la tasa de deforestación en el estado de Tabasco. Kuxulkab’. Revista de la División Académica de Ciencias Biológicas Universidad Juárez Autónoma de Tabasco, 2008. Disponible en: http://www.dgbiblio.unam.mx Acceso el 15 de diciembre de 2017.

[19] 19. Malik A, Abdalla R. Mapping the impact of air travelers on the pandemic spread of (H1N1) influenza. Model Earth Syst Environ. 2016;2:91. 10.1007/s40808-016-0147-1

[20] 20. Fithian W, Hastie T. Finite-sample equivalence of several statistical models for presence-only data. Ann App Stat. 2013;7(4):1917-39. DOI: 10.1214/13-AOAS667 10.1214/13-AOAS667 PMC4258396 25493106

[21] 21. Merow C, Smith M, Silander J. A practical guide to MaxEnt for modeling species’ distributions: what it does, and why inputs and settings matter. Ecography. DOI: 10.1111/j.1600-0587.2013.07872.x

[22] 22. Escobar LE. Modelos de nicho ecológico en salud pública: cinco preguntas cruciales. Rev Panam Salud Publica. 2016;40(2). Disponible en: http://iris.paho.org/xmlui/handle/123456789/31163 27982364

[23] 23. Xu M, Cao C, Wang D, Kan B. Identifying environmental risk factors of cholera in a coastal area with geospatial technologies. Int J Environ Res Public Health. 2014;12(1),354-70. DOI: 10.3390/ijerph120100354 10.3390/ijerph120100354 PMC4306866 25551518

[24] 24. Soberón J, Nakamura M. Niches and distributional areas: concepts, methods and assumptions. PNAS. 2009;106S2):19644-50. DOI: 10.1073/pnas.0901637106 10.1073/pnas.0901637106 PMC2780935 19805041

[25] 25. BIOCLIM^®^ WorldClim-Global Climate Data. Free climate data for ecological modeling and GIS Disponible en: http://www.worldclim.org/

[26] 26. Qun Fong L, Xin-Low L, Yin-Lui L, Hong-Wu Y, Song L, Yang Y, et al. Mapping spread and risk of avian influenza A (H7N9) in China. Sci Rep. 2013;3. DOI: 1038/srep02722. 10.1038/srep02722 PMC3784030 24072008

[27] 27. Prosser DJ, Hungerford LL, Erwin RM, Ottinger MA, Takekawa JY, Newman SH, et al. Spatial modeling of wild bird risk factors for highly pathogenic A (H5N1) avian influenza virus transmission. Avian Dis. 2016;60(S1):329-36. DOI: 10.1637/11125-050615-Reg 10.1637/11125-050615-Reg 27309075

[28] 28. Kranstauber B, Weinzierl R, Wikelski M, Safi K. Global aerial flyways allow efficient travelling. Ecol Lett. 2015;18(12):1338-45. 10.1111/ele.12528 26477348

[29] 29. Kemp MU, Shamoun-Baranes J, Dokter AM, Van Loon E, Bouten W.The influence of weather on the flight altitude of nocturnal migrants in mid-latitudes. Ibis. 2013;155:734-49. DOI: 10.1111/ibi.12064

[30] 30. Colemin JP. 2009. Autocorrelación espacial e indicadores locales de asociación espacial. Importancia, estructura y aplicación. Rev Univ Geogr. 2009;18(1).

